# Supplementary material for: Prediction of the gene expression in normal lung tissue by the gene expression in blood
Source: BMC Med Genomics. 2015 Nov 17;8:77. doi: 10.1186/s12920-015-0152-7 (PMC4650316; doi:10.1186/s12920-015-0152-7)
Supplement: Additional file 1: — Table S1. Genes whose expression in lung is predicted by the gene expression in blood in multivariate linear regression model with gene expression in lung as outcome, gene expression in blood, age and gender as predictors. We included all nominally significant (type1 error ≤0.05) genes. Entrez gene IDs are shown. Table S2. Genes whose expression in lung is significantly (P ≤ 0.05) predicted by age in multivariate linear regression model with gene expression in lung as outcome, gene expression in blood, age and gender as predictors. Table S3. Genes whose expression in lung is significantly (P ≤ 0.05) predicted by gender in multivariate linear regression model with gene expression in lung as outcome, gene expression in blood, age and gender as predictors. [file 12920_2015_152_MOESM1_ESM.pdf]

PREDICTION OF THE GENE EXPRESSION IN NORMAL LUNG TISSUE BY THE GENE EXPRESSION IN BLOOD

Justin W Halloran[1], Dakai Zhu[2], David C Qian[2], Jinyoung Byun[2], Olga Y Gorlova[2], Christopher I Amos[2], and Ivan P Gorlov[2]*

* Corresponding author: Ivan P Gorlov ivan.p.gorlov@dartmouth.edu

1 Department of Biological Sciences, Dartmouth College, 78 College St., Hanover, NH 03755, USA

2 Department of Biomedical Data Science, The Geisel School of Medicine, Dartmouth College, HB7937, One Medical Center Dr., Dartmouth-Hitchcock Medical Center, Lebanon, NH 03756, USA

Email: Justin W Halloran Justin.W.Halloran.16@dartmouth.edu - Dakai Zhu Dakai.Zhu@dartmouth.edu - David C Qian David.C.Qian.GR@dartmouth.edu - Jinyoung Byun Jinyoung.Byun@dartmouth.edu - Olga Y Gorlova Olga.Y.Gorlova@dartmouth.edu - Christopher I Amos Christopher.I.Amos@dartmouth.edu – Ivan P Gorlov Ivan.P.Gorlov@dartmouth.edu

ABSTRACT

Background

Comparative analysis of gene expression in human tissues is important for understanding the molecular mechanisms underlying tissue-specific control of gene expression. It can also open an avenue for using gene expression in blood (which is the most easily accessible human tissue) to predict gene expression in other (less accessible) tissues, which would facilitate the development of novel gene expression based models for assessing disease risk and progression. Until recently, direct comparative analysis across different tissues was not possible due to the scarcity of paired tissue samples from the same individuals.

Methods

In this study we used paired whole blood/lung gene expression data from the Genotype-Tissue Expression (GTEx) project. We built a generalized linear

regression model for each gene using gene expression in lung as the outcome and gene expression in blood, age and gender as predictors.

Results

For ~18% of the genes, gene expression in blood was a significant predictor of gene expression in lung. We found that the number of single nucleotide polymorphisms (SNPs) influencing expression of a given gene in either blood or lung, also known as the number of quantitative trait loci (eQTLs), was positively associated with efficacy of blood-based prediction of that gene's expression in lung. This association was strongest for shared eQTLs: those influencing gene expression in both blood and lung.

Conclusions

In conclusion, for a considerable number of human genes, their expression levels in lung can be predicted using observable gene expression in blood. An abundance of shared eQTLs may explain the strong blood/lung correlations in the gene expression.

Keywords: gene expression; normal lung tissue; normal blood; Genotype-Tissue Expression Project; GTEx

Background

Study of tissue specificity in gene expression is important for understanding tissue biology and can facilitate an identification of genes associated with risk of human diseases [1]. A number of studies on comparative analysis of the gene expression in different tissues were published [2-4]. Pan-tissue analysis of the gene expression has shown that, on the genome level, there is a strong positive correlation in expression, meaning that genes expressed at a low level tend to have low expression across multiple tissues [5-7].

Correlation on the gene level, e.g. correlation between expression of a given gene in blood and in lung tissue across different individuals, is poorly studied. The major reason for this is lack of paired data: samples from the different tissues of the same individual. Such samples were not available until very recently. Genotype-Tissue Expression Project (GTEx), launched by the NIH in

2010, has yielded paired gene expression data, allowing analysis of the gene expression in different tissues originating from the same individual.

Analysis of the inter-tissue correlations, especially between gene expression in blood and other tissues, has obvious practical significance. Blood is the most accessible human tissue and identification of the genes whose expression in blood mirrors the gene expression in other tissue may be important for cancer risk prediction based on the gene expression profiling in blood.

The goal of this study was: (i) To test the hypothesis that for some human genes, gene expression in blood mirrors the gene expression in lung; and (ii) To identify gene characteristics influencing blood/lung correlations in expression.

RESULTS

Gene expression in blood mirrors gene expression in lung

At the genome level (mean expression level of the gene in blood versus the mean expression level in lung), there was a strong positive correlation: Spearman's rank correlation coefficient (SRCC) = 0.90, N = 22,704, P = $2 \cdot 10^{-34}$. Figure 1 shows the scatterplot of the mean expression in lung versus mean expression in blood. At the gene level (correlation between paired blood/lung samples), average SRCC was positive and significant: average SRCC = 0.06, N = 22,704, P = $3.1 \cdot 10^{-8}$. P-value is shown for the testing null hypothesis that mean SRCC equals zero.

Gene specific linear regression models

A total of 22,704 gene-specific generalized linear regression models were built with gene expression in lung tissue as outcome and gene expression in blood, age and gender as predictors. For 3,671 genes, at least one predictor was nominally significant. For 1,216 genes, age was the only significant predictor; for 331 genes, gender was the only significant predictor; and for 1,748 genes, gene expression in blood was the only significant predictor of the gene expression in lung. The list of genes whose expression in lung can be predicted by age, gender or expression level in blood can be found in supplementary materials.

Number of eQTLs and prediction efficacy

Positive correlation between gene expression in blood and the gene expression in lung can be driven by shared genetic polymorphisms influencing gene expression – eQTLs. According to GTEx, there are 235,576 eQTLs for blood and 222,038 for lung tissue. The number of eQTLs per gene varied from 0 to several hundred, e.g. MICA has 539 blood and 482 lung eQTLs. More than 80% (189,869) of blood eQTLs also influence gene expression in lung (shared eQTLs). For 99.9% of shared eQTLs the direction of the effect is the same in blood and lung, meaning that an allele that increases gene expression in blood also increases gene expression in lung. Therefore, more than 80% of eQTLs are shared by blood and lung tissues and, even more importantly, essentially all of them have the same direction of the effect on gene expression.

We found significant positive correlation between the number eQTLs for the gene and β_{blood}: SRCC = 0.08, N =22,704, P = $1.6x10^{-12}$. Number of eQTLs for the gene does not correlate with β_{blood} neither for age: SRCC = -0.006, N =22,704, P = 0.49, or gender: SRCC = -0.01, N =22,704, P = 0.28. Correlation of β_{blood} with the number of eQTLs for lung also was significant: SRCC = 0.1, N =22,704, P = $3.7x10^{-14}$. For the shared eQTLs the correlation between β_{blood} and the number of eQTLs was highest: SRCC = 0.14, N =22,704, P = $7.6x10^{-19}$.

eQTLs and inter-individual variation in expression

eQTLs can contribute to inter-individual variation in the gene expression. We divided all genes into 4 groups based on the number of the reported eQTLs: (1) no reported eQTLs; (2) 1 to 10 eQTLs; (3) 10 to 51; and (4) 52 or more. Variance was estimated in each group separately for lung and blood tissues (Figure 2). The variance was highest in the 4th group. For both tissue types, there was a significant positive association between the number of eQTLs and inter-individual variance in the gene expression.

Gene characteristics associated with the blood-based prediction efficacy

We tried to determine if factors other than number of eQTLs gene characteristics influence efficacy of the blood-based prediction of the gene expression in lung. We used the same gene characteristics as we used in our recently published

paper on prediction of SNP reproducibility [8]. Table 1 shows the list of analyzed characteristics and corresponding statistics. For information about sources of the data we refer the reader to the original publication [8]. Gene characteristics significantly associated with prediction efficacy of the gene expression in lung based on the gene expression in blood include nuclear localization, acetylation, methylation, ubiquitination, and the size of the coding region.

DISCUSSION

Gene expression is controlled by a combination of general and tissue-specific transcription factors (TFs). Transcription initiation requires a formation of a multi-protein complex that includes general transcription factors (GTFs) [9]. GTFs are not tissue-specific: binding of GTFs to the promoter regions is required for initiation of transcription of the gene in any tissue. GTFs bind the promoter region of the gene in the sequence-specific manner and the binding efficacy is sequence-dependent [10]. Genetic polymorphisms in the GTFs' binding sites modulate gene expression similarly across different tissue types. Tens of thousands of SNPs were detected in promoter regions of the human genes [11]. In fact, SNP density in regulatory regions of the human genes is higher compared to other regions [12], which can be explained by a higher G+C content in the regulatory regions. A higher G+C content is associated with elevated mutation rate, especially in CpG sites [13, 14]. Promoter regions are not the only sites harboring regulatory elements modulating gene expression. 5'UTR regions often contain regulatory elements, not to mention the fact that there are long distance regulatory elements acting throughout DNA looping. Many of them are evolutionarily conserved, and may act through regulation of initial stages of transcription [15, 16].

Our recent (February 25th, 2015) access of dbSNP database indicated that there were almost 400K SNPs in 5'UTRs. The existence of multiple polymorphic sites in the human genome modulating gene expression level is supported by eQTL analyses. The first eQTL study based on the analysis of lymphoblastoid cell lines

and a rather limited number of SNPs generated by HapMap project [17] has identified almost 52K eQTLs with FDR<0.1.

Our analysis demonstrated that: (i) for about 18% of the human genes, the expression in lung can be predicted by an assessment of the gene expression in blood, and (ii) the efficacy of the prediction depends on the number of shared eQTLs. Different human tissues are expected to have exactly the same set of eQTLs, since they are the same (except somatic mutations) on the DNA level. However, functional effect of eQTLs can be tissue-specific because transcription factors are often tissue-specific.

We used P-values that were not adjusted for multiple testing. This suggests that some nominally significant correlations can be false-positives. Among 16,998 assessed genes the number of nominally significant blood/lung gene expression correlations was 2,380. The expected number of correlations based on the type 1 error of 0.05 is 850 which is lower than the observed number ($\chi^2 = 793.5, df = 1, p = 1.2^{-56}$). Though this result clearly indicates the presence of true positives, we need to be cautious about using them to define the list of genes in the human genome whose expression in lung can be predicted by gene expression in blood. A comprehensive list of such genes cannot be generated using available data because the sample size is too small, and data on tobacco smoke exposure are lacking (see the study limitations section). The goal of this study was to demonstrate that (i) for a considerable number of human genes, their expression in lung could be predicted by the level of their expression in blood, and (ii) to identify factors influencing blood/lung correlations.

We found that the majority of eQTLs are shared and that an absolute majority of shared eQTLs has the same direction of effect in different tissues, meaning that if a given allele increases the expressions of the gene in blood, it also will increase the expression of the same gene in lung. One can expect that an individual with an excess of up-regulating eQTLs will have elevated expression of the gene across different tissues.

In univariate analyses eQTLs were the most significant predictors of blood/lung correlation in gene expression. We found that nuclear localization,

posttranslational modification of the gene product, and gene size are associated with the efficacy of the blood-based prediction of the gene expression in lung. We used a multivariable linear regression model to test if those characteristics are eQTL-independent. Minus LOG(P) for the statistical significance of the β for the gene expression in blood was used as outcome, and number of shared eQTLs, nuclear localization, gene size and posttranslational protein modification were used as predictors. Only number of eQTLs remained significant in the model: $F = 336.5$, $df = 1$, $P = 3.6 \times 10^{-16}$ while nuclear localization, posttranslational protein modifications and gene size were not significant: corresponding P-values are 0.25, 0.19 and 0.06.

The results of this analysis suggest that shared eQTLs with the same direction of the effect drive blood/lung correlation in gene expression. Based on this observation, one can expect similar correlations for other tissue types. Unfortunately, GTEx database does not have enough paired samples to explore correlations between other tissues.

Study limitations

The conducted study has two major limitations.

A small sample size. GTEx data are unique since they provide gene expression in normal human tissues from the same individual. However, the number of available samples is relatively small (31 paired blood/lung samples). This suggests that the statistical power to detect statistically significant correlations between gene expression in blood and lung is limited. One can expect that the real number of genes whose expression in blood reflects their expression in lung is higher.

Lack of data on environmental exposures (smoking). Exposure to tobacco smoke has been shown to influence gene expression in lung [18, 19] and blood [20]. Smoking data for GTEx participants were not available at the moment of the analysis. It is difficult to predict the effect of smoking on blood/lung correlation in gene expression: for genes whose expression in blood and lung changes similarly in response to tobacco smoke exposure, the correlation can be stronger, while the correlation is expected to be weaker for genes that are tobacco smoke

sensitive in one tissue but not in the other. The results of the conducted analysis, hovever, suggest that genetic (eQTLs) rather than environmental (tobacco smoke) factors are major drivers of the positive association between blood and lung expression.

In conclusion we found that for about 20% of the genes, expression level in lung can be predicted by assessment of the expression level in blood. Genes with a strong genetic component in the control of the expression level (number of eQTLs) show stronger blood/lung association in gene expression.

In conclusion, the results of the conducted analysis also indicate that the expression levels of the genes with a strong genetic component in the control of the expression show a higher inter-individual variation in expression level. We believe that genes whose expression in lung can be predicted by assessment of the gene expression in blood can be a valuable resource for blood-based disease risk prediction models.

MATERIALS AND METHODS

All data used in this study were acquired from publicly available sources and we have not conducted any research on human subjects/samples ourselves.

Data sources

We used GTEx data http://www.gtexportal.org/home/ to correlate gene expression in blood with the expression level of the same gene in lung from the same donor. For the correlation analysis we used non-parametric Spearman's rank test because for many genes the distribution of expression values deviates from the normal distribution. A total of 31 donors were identified for which pre-mortal gene expression in whole blood and paired gene expression in normal lung is available. Donors with a history of lung or respiratory conditions (asthma and pneumonia) were excluded from the analysis. We used lung tissue because it has the largest number of paired samples. Table 2 shows basic demographic characteristics of the donors. Gene expressions in both blood and lung tissues were assessed by RNA sequencing with median sequencing depth of 82.1

million mapped reads per sample. Details on sequencing and data processing can be found in recent GTEx publication[21].

eQTLs

We used data on lung and blood eQTLs recently released by GTEx. eQTLs with reported statistical significance level $<10^{-5}$ were used in the analysis. eQTLs were subdivided into 3 categories: (1) Blood eQTLs – those influencing the expression level of the gene in blood tissue; (2) Lung eQTLs – those influencing the expression level of the gene in lung; and (3) Shared eQTLs – those influencing the expression level of the gene in blood and lung.

Efficacy of the prediction of the gene expression in lung based on the gene expression in blood

To predict gene expression in lung, we built gene-specific linear regression models with gene expression in lung as outcome and the gene expression in blood, age, and gender as predictors. Model coefficients for predicting variables were used to assess an efficacy of the prediction of the gene expression in lung based on the gene expression blood. We used $-LOG(P_{blood})$: where P_{blood} is type one error for the difference of regression coefficient from 0 (null hypothesis). We did not use correlation coefficient because, for some genes, the correlation between gene expression in blood and lung was driven by gender or age-related effects.

Gene characteristics influencing correlation between gene expression in blood and lung

We tested the hypothesis that prediction efficacy of the gene expression in lung based on the gene expression in blood depends on gene characteristics. We used a set of the gene characteristics from our recently published paper on prediction of SNP reproducibility in GWASs [8].

Statistical analysis

Data analysis was performed in R 3.0.2 (The R Foundation for Statistical Computing). Normalized expression data was obtained from Affymetrix expression data from GTEx at

http://www.ncbi.nlm.nih.gov/geo/query/acc.cgi?acc=GSE45878 for 22,704 genes. A linear regression model was built for every gene using the “lm” function in the base R package, using expression levels in blood, and the age and gender of each individual into account as predictors.

For identification of gene characteristics associated with efficacy of prediction of the gene expression in lung based on expression level in blood, we used a nonparametric Kolmogorov–Smirnov test for binary traits, and a Spearman’s rank correlation for non-binary traits.

Abbreviations

GTEx: Genotype-Tissue Expression project

eQTLs: Expressed quantitative trait loci

SNP: Single nucleotide polymorphism

SRCC: Spearman’s rank correlation coefficient

Competing interests

The authors declare that they have no competing interests.

Authors’ contributions

IPG, CIA, and OYG designed the method and experiments. JWH, DZ, DCQ, JB and IPG carried out the computation and FSV performed the analysis. IPG drafted the manuscript. CIA and OYG provided advice and suggestions, and revised the manuscript. All authors read and approved the final manuscript.

Additional file

Additional file 1: Table S1. Genes whose expression in lung is predicted by the gene expression in blood in multivariate linear regression model with gene expression in lung as outcome, gene expression in blood, age and gender as predictors. We included all nominally significant (type1 error ≤0.05) genes. Entrez gene IDs are shown. **Table S2.** Genes whose expression in lung is significantly (P≤0.05) predicted by age in multivariate linear regression model with gene expression in lung as outcome, gene expression in blood, age and gender as predictors. **Table S3.** Genes whose expression in lung is significantly (P≤0.05) predicted by gender in multivariate linear regression model with gene expression in lung as outcome, gene expression in blood, age and gender as predictors.

Acknowledgements

This work was supported in part by the National Institutes of Health U19 CA148127 grant and the National Institutes of Health Grants 5 P30 CA016672, LM009012, LM010098 and GM103534. The funders had no role in study design, data collection and analysis, decision to publish, or preparation of the manuscript.

REFERENCES

1. Greene CS, Krishnan A, Wong AK, Ricciotti E, Zelaya RA, Himmelstein DS, Zhang R, Hartmann BM, Zaslavsky E, Sealfon SC *et al*: **Understanding multicellular function and disease with human tissue-specific networks**. *Nature genetics* 2015.
2. Sinha P, Singh VK, Suryanarayana V, Krishnamurthy L, Saxena RK, Varshney RK: **Evaluation and Validation of Housekeeping Genes as Reference for Gene Expression Studies in Pigeonpea (Cajanus cajan) Under Drought Stress Conditions**. *PloS one* 2015, **10**(4):e0122847.
3. Eisenberg E, Levanon EY: **Human housekeeping genes, revisited**. *Trends in genetics : TIG* 2013, **29**(10):569-574.
4. Liu X, Yu X, Zack DJ, Zhu H, Qian J: **TiGER: a database for tissue-specific gene expression and regulation**. *BMC bioinformatics* 2008, **9**:271.
5. Axelsen JB, Lotem J, Sachs L, Domany E: **Genes overexpressed in different human solid cancers exhibit different tissue-specific expression profiles**. *Proceedings of the National Academy of Sciences of the United States of America* 2007, **104**(32):13122-13127.
6. Chiang AW, Shaw GT, Hwang MJ: **Partitioning the human transcriptome using HKera, a novel classifier of housekeeping and tissue-specific genes**. *PloS one* 2013, **8**(12):e83040.
7. Jacox E, Gotea V, Ovcharenko I, Elnitski L: **Tissue-specific and ubiquitous expression patterns from alternative promoters of human genes**. *PloS one* 2010, **5**(8):e12274.
8. Gorlov IP, Moore JH, Peng B, Jin JL, Gorlova OY, Amos CI: **SNP characteristics predict replication success in association studies**. *Human genetics* 2014, **133**(12):1477-1486.
9. Latchman DS: **Transcription factors: an overview**. *The international journal of biochemistry & cell biology* 1997, **29**(12):1305-1312.
10. Kornberg RD: **The molecular basis of eukaryotic transcription**. *Proceedings of the National Academy of Sciences of the United States of America* 2007, **104**(32):12955-12961.
11. Tahira T, Baba S, Higasa K, Kukita Y, Suzuki Y, Sugano S, Hayashi K: **dbQSNP: a database of SNPs in human promoter regions with allele frequency information determined by single-strand conformation polymorphism-based methods**. *Human mutation* 2005, **26**(2):69-77.

12. Guo Y, Jamison DC: **The distribution of SNPs in human gene regulatory regions**. *BMC genomics* 2005, **6**:140.
13. Koeberl DD, Bottema CD, Buerstedde JM, Sommer SS: **Functionally important regions of the factor IX gene have a low rate of polymorphism and a high rate of mutation in the dinucleotide CpG**. *American journal of human genetics* 1989, **45**(3):448-457.
14. Walser JC, Ponger L, Furano AV: **CpG dinucleotides and the mutation rate of non-CpG DNA**. *Genome research* 2008, **18**(9):1403-1414.
15. He C, Wang X, Zhang MQ: **Nucleosome eviction and multiple co-factor binding predict estrogen-receptor-alpha-associated long-range interactions**. *Nucleic acids research* 2014, **42**(11):6935-6944.
16. Kulaeva OI, Nizovtseva EV, Polikanov YS, Ulianov SV, Studitsky VM: **Distant activation of transcription: mechanisms of enhancer action**. *Molecular and cellular biology* 2012, **32**(24):4892-4897.
17. Cheung VG, Spielman RS, Ewens KG, Weber TM, Morley M, Burdick JT: **Mapping determinants of human gene expression by regional and genome-wide association**. *Nature* 2005, **437**(7063):1365-1369.
18. Spira A, Beane J, Shah V, Liu G, Schembri F, Yang X, Palma J, Brody JS: **Effects of cigarette smoke on the human airway epithelial cell transcriptome**. *Proceedings of the National Academy of Sciences of the United States of America* 2004, **101**(27):10143-10148.
19. Sridhar S, Schembri F, Zeskind J, Shah V, Gustafson AM, Steiling K, Liu G, Dumas YM, Zhang X, Brody JS *et al*: **Smoking-induced gene expression changes in the bronchial airway are reflected in nasal and buccal epithelium**. *BMC genomics* 2008, **9**:259.
20. Charlesworth JC, Curran JE, Johnson MP, Goring HH, Dyer TD, Diego VP, Kent JW, Jr., Mahaney MC, Almasy L, MacCluer JW *et al*: **Transcriptomic epidemiology of smoking: the effect of smoking on gene expression in lymphocytes**. *BMC medical genomics* 2010, **3**:29.
21. Consortium GT: **Human genomics. The Genotype-Tissue Expression (GTEx) pilot analysis: multitissue gene regulation in humans**. *Science* 2015, **348**(6235):648-660.

Figure legends:

Figure 1. The scatterplot of the mean expression in blood versus mean expression in lung. Each dot represents a gene.

Figure 2. Inter-individual variance in expression level in the genes stratified by the reported number of eQTLs. Left panel shows the variance of the gene

expression level in blood and the right panel shows the variance in gene expression level in lung in four groups of genes stratified by the number of the reported eQTLs.

Table 1. Gene characteristics used in the analysis to estimate if they are associated with efficacy of prediction of gene expression in lung based on gene expression in blood.

Trait	Statistic	P-Value	Method
Nuclear localization of the gene product	0.04	0.00001	Kolmogorov-Smirnov
Size of the gene region in nucleotides	4.37	0.00009	Spearman's
Number of exons in the gene	5.92	0.00521	Spearman's
PTM* (ubiquitination)	0.02	0.00573	Kolmogorov-Smirnov
PTM (acetylation)	0.03	0.01269	Kolmogorov-Smirnov
PTM (methylation)	0.04	0.01735	Kolmogorov-Smirnov
Plasma membrane localization of the gene product	0.03	0.08340	Kolmogorov-Smirnov
Receptor	0.03	0.10019	Kolmogorov-Smirnov
Signal transducer	0.03	0.12617	Kolmogorov-Smirnov
Nuclear membrane localization	0.09	0.12746	Kolmogorov-Smirnov
Kinase	0.06	0.17557	Kolmogorov-Smirnov
Extracellular localization	0.03	0.20352	Kolmogorov-Smirnov
Growth factor	0.09	0.22230	Kolmogorov-Smirnov
Phosphatase	0.10	0.23498	Kolmogorov-Smirnov
PTM (sumoylation)	0.06	0.24742	Kolmogorov-Smirnov
Conservation Index of the gene	4.56	0.31737	Spearman's
Secreted gene product	0.02	0.34382	Kolmogorov-Smirnov
Transcription factor	0.02	0.39715	Kolmogorov-Smirnov
PTM (phosphorylation)	0.01	0.61248	Kolmogorov-Smirnov
Housekeeping gene	0.03	0.68928	Kolmogorov-Smirnov
Tissue specific gene expression	0.01	0.76322	Kolmogorov-Smirnov
Cytoplasmic localization of the gene product	0.01	0.92317	Kolmogorov-Smirnov

* PTM – Post Translational Modifications

Table 2. Basic demographic characteristics of the donors used for the prediction of the gene expression level in lung based on the gene expression level in blood

	Age group				
	20-29	30-39	40-49	50-59	60-69
Female	5	1	4	4	3
Male	2	3	3	3	3
Mean ischemic time	-4.5	-6.8	-5.1	-6.2	-4.9

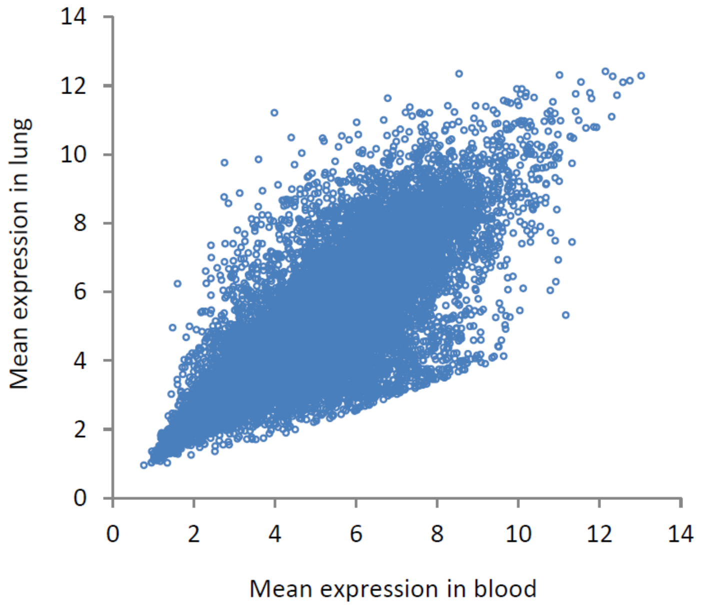

14
12
10
8
6
4
2
0
0
2
4
6
8
10
12
14
Mean expression in lung
Mean expression in blood

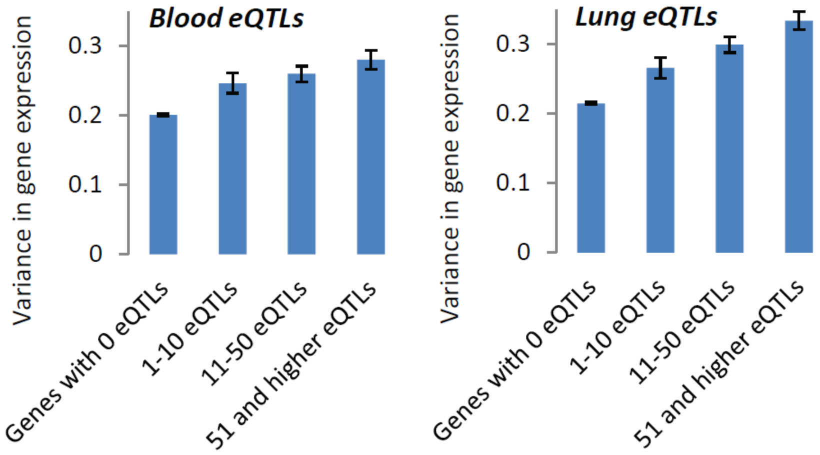
Blood eQTLs
Lung eQTLs
Variance in gene expression
0.3
0.2
0.1
0
Genes with 0 eQTLs
1-10 eQTLs
11-50 eQTLs
51 and higher eQTLs
